# Differential expression analysis of miRNAs in macrophage-derived exosomes in the tuberculosis-infected bone microenvironment

**DOI:** 10.3389/fmicb.2023.1236012

**Published:** 2023-08-03

**Authors:** Zhicheng Sun, Xiaoyang Pang, Xiyang Wang, Hao Zeng

**Affiliations:** ^1^Department of Spinal Surgery, Xiangya Hospital of Central South University, Changsha, Hunan, China; ^2^Department of Spinal Surgery, The First Affiliated Hospital of Guangxi Medical University, Nanning, Guangxi, China; ^3^Guangxi Key Laboratory of Regenerative Medicine, Nanning, Guangxi, China

**Keywords:** spinal tuberculosis, bone microenvironment, macrophages, exosomes, miRNA-seq, biomarker

## Abstract

**Background:**

Macrophages play an important role in regulating the course of spinal tuberculosis within the bone microenvironment. This study aimed to investigate the differential expression of miRNA in macrophage-derived exosomes within the tuberculosis-infected bone microenvironment, to identify miRNAs that hold potential as diagnostic markers and therapeutic targets.

**Methods:**

We established study cohorts for spinal tuberculosis, collected bone marrow blood samples, isolated macrophage exosomes, and performed exosome miRNA sequencing. A miRNA-mRNA co-expression network was constructed using WGCNA analysis. Gene GO analysis and KEGG pathway enrichment analysis were performed using KOBAS software. Target miRNAs were selected based on fold change, *P*-value, and false discovery rate, and their validation was carried out using qRT-PCR and ROC curve studies. Subsequently, we constructed a target gene network for these miRNAs and performed KEGG pathway enrichment analysis to explore the potential signaling mechanisms involved in regulating the disease course of spinal tuberculosis.

**Results:**

Our findings revealed that macrophages from the tuberculosis-infected bone microenvironment exhibited an M1 phenotype. The successful extraction of exosomes from macrophage supernatants was confirmed through electron microscopy, particle size analysis, and protein blot analysis. Exosome miRNA-seq demonstrated that 28 miRNAs were up-regulated, while 34 miRNAs were down-regulated in individuals with spinal tuberculosis. GO analysis and KEGG pathway enrichment analysis indicated that the differentially expressed miRNAs were involved in various biological processes, cell components, molecular functions, and signaling pathways, which collectively contribute to the regulation of the disease course of spinal tuberculosis. Notably, miRNA-125b-5p was successfully selected based on fold change, *p*-value, and false discovery rate. qRT-PCR validation further confirmed the significant up-regulation of miRNA-125b-5p in spinal tuberculosis. The ROC curve revealed that miR-125b-5p is a potential diagnostic biomarker for spinal tuberculosis. Moreover, construction of the miRNA-125b-5p target gene network and subsequent KEGG enrichment analysis highlighted the importance of MAPK, TNF, Ras, Rap1, and the PI3K-Akt signaling pathways in the regulation of the disease course of spinal tuberculosis.

**Conclusion:**

Our study demonstrates differential expression of miRNAs in macrophage-derived exosomes in the tuberculosis-infected bone microenvironment. Specifically, MiRNA-125b-5p is significantly up-regulated in spinal tuberculosis and shows potential as a diagnostic biomarker for spinal tuberculosis.

## 1. Introduction

*Mycobacterium* tuberculosis (MTB) infection affects approximately one-third of the global population, with 11 million new cases reported annually, including 150,000 cases of spinal tuberculosis (STB) (Pang et al., 2019). The onset of STB is insidious and difficult to diagnose in the early stages. As the disease progresses, it often leads to spinal instability, kyphosis, and severe neurological complications, including paralysis (Khanna and Sabharwal, 2019), severely affecting patients’ quality of life.

Macrophages play an important regulatory role in the regulation of tuberculosis. They act as the first line of defense against *Mycobacterium* tuberculosis, actively phagocytosing and eliminating the pathogen through cytocytosis and lysosomal degradation (Sachdeva et al., 2020). Additionally, macrophages act as antigen-presenting cells, degrading *Mycobacterium* tuberculosis into immunogenic antigenic polypeptides presented to CD4 + T cells and CD8 + T cells via MHC I and II molecules, triggering an adaptive immune response and activating effector cell killing mechanisms to eliminate the pathogen (Cui et al., 2020). Simultaneously, *Mycobacterium* tuberculosis can modulate macrophage function to maintain a dynamic balance between host protection and tissue damage (Genoula et al., 2020).

Exosomes, membrane-bound structures derived from cells, are heterogeneous in nature and range from 30 to 150 nm in diameter. They contain complex RNAs and proteins that serve as important mediators of cellular functions (Liu Y. et al., 2020). Exosomal miRNAs are important epigenetic regulators that exhibit differential expression in tuberculosis and other infectious diseases. Alipoor et al. (2019) demonstrated significantly elevated expression of miR-484, miR-425, and miR-96 in the serum of patients with pulmonary tuberculosis, correlating with tuberculosis activity. Lyu et al. (2019) revealed distinct exosomal miRNA expression profiles in peripheral blood serum between patients with tuberculosis, latent stage tuberculosis, and healthy controls. Similarly, miRNA expression changes are implicated in the regulation of macrophage metabolism and function. Sequencing of exosomal miRNAs in *Mycobacterium* tuberculosis-infected peripheral blood mononuclear macrophages showed significant differential expression of several miRNAs, with most of them involved in cellular metabolic processes (Alipoor et al., 2017).

Previous studies have emphasized the significance of macrophage-derived exosomes in infectious diseases. Infected macrophages can release exosomes that induce uninfected macrophages to produce pro-inflammatory factors, thereby amplifying the inflammatory response (Imamiya et al., 2023). Furthermore, macrophage-derived exosomes possess antigen-presenting and immunomodulatory functions (Smith et al., 2017). However, the role of macrophage-derived exosomes in the tuberculosis-infected bone microenvironment remains poorly understood, and their involvement in regulating the course and metabolic processes of spinal tuberculosis has not been explored. In this study, we investigated the differential miRNA expression in macrophage-derived exosomes from the tuberculosis-infected bone microenvironment using high-throughput sequencing. Our objective was to identify miRNAs with potential diagnostic value and therapeutic promise, thereby laying the foundation for further research on miRNA mechanisms.

## 2. Materials and methods

### 2.1. Study subjects and biological samples

This study was approved by the Ethics Committee of Xiangya Hospital, and all patients provided informed consent before surgery. Ten patients each with spinal tuberculosis and disc degeneration, who were surgically treated at our hospital between January 2019 and June 2022, were selected and divided into two groups: the spinal tuberculosis (STB) group and the non-spinal tuberculosis (NSTB) group. Intraoperatively, bone marrow blood samples were collected from the pedicle screw channel of the affected vertebral body in STB patients and from the fixed segment in NSTB patients.

For miRNA sequencing analysis, three bone marrow blood samples were selected from each group. All ten bone marrow blood samples from each group were used for qRT-PCR validation analysis of differentially expressed miRNA. Additionally, bone marrow blood samples were collected from twenty preoperatively diagnosed spinal infection patients, clinically suspected of having spinal tuberculosis during the same period. Differentially expressed miRNA expression levels were examined to establish ROC curves.

#### 2.1.1. Methods of bone marrow blood collection

**FIGURE 1:**
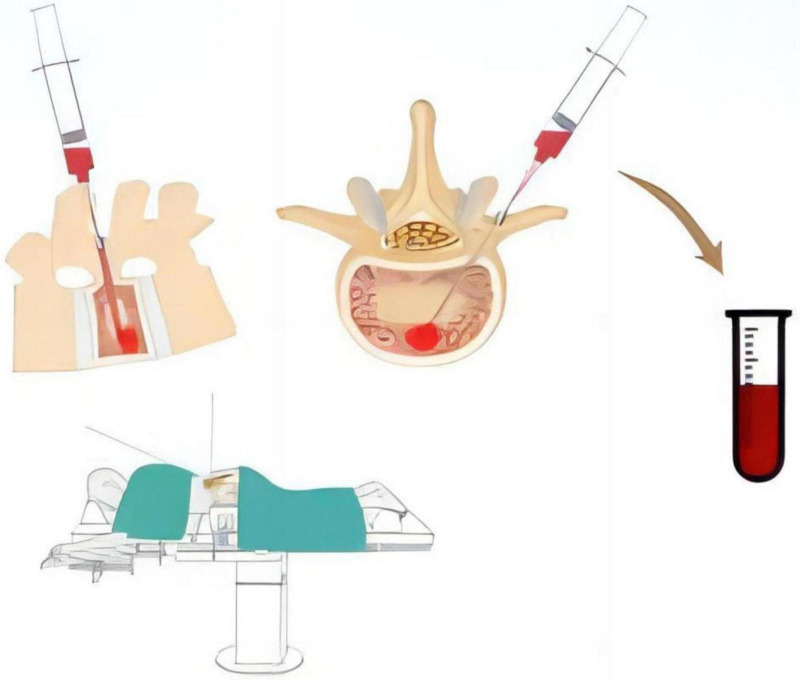
Schematic representation of the method for collecting bone marrow blood samples from the constructed pedicle screw channel during the procedure.

All patients underwent surgery in the prone position using a pure posterior approach. The channel for pedicle screw implantation was identified using various surgical instruments, and peripheral blood around the channel entry point was aspirated throughout the procedure. A 20 ml syringe was used to aspirate 15 ml of bone marrow blood from the depth of the channel, which was then reserved for further analysis (Figure 1).

To ensure the reliability of the sequencing results and minimize potential confounding, we established strict inclusion criteria as follows: ➀ The STB and NSTB groups were required to meet the diagnostic criteria for spinal tuberculosis and degenerative disc disease, respectively. ➁ All patients had to meet the surgical indications, and intraoperative construction of the pedicle screw channel was required. ➂ The age range was limited to 20–40 years, and female patients in the perimenopausal period were excluded. ➃ Patients with a history of paralysis, trauma (especially to the spine, pelvis, and lower extremities), or surgery resulting in prolonged bed rest or significant limitation of daily activities within the past 6 months were excluded. ➄ Patients with a history of other infectious bone and joint diseases, metabolic diseases, cancer, or chronic diseases were excluded. ➅ Patients with a history of psychiatric disorders were also excluded.

### 2.2. Isolation of bone marrow blood mononuclear cells

Bone marrow blood samples were mixed with an equal volume of serum-free buffer containing 5 IU/ml heparin to obtain a cell suspension. The suspension was then mixed with an equal volume of lymphocyte separation solution and centrifuged at 500 × *g* for 20 min at room temperature. The upper plasma was aspirated, and the mononuclear cells at the interface of the plasma layer and the lymphocyte separation solution were collected. Subsequently, a twofold volume of Hanks’ solution containing 5 IU/ml heparin and 2% inactivated calf serum was added to the collected cells. The mixture was then mixed and centrifuged at 200 × *g* for 10 min to remove the platelets retained in the cell suspension. The cells were washed twice with the same Hanks solution and centrifuged at 500 × *g* for 10 min each time to wash away the lymphocyte separation solution. Finally, the cell concentration was adjusted to 2*10^^9^/L using a cell culture medium containing 10% calf serum, and the cells were kept in reserve.

### 2.3. Macrophage induction, purification, and identification

#### 2.3.1. Macrophage induction

The isolated mononuclear cells were adjusted to 1*10^^5^/ml using 20% FBS/DMEM medium supplemented with 30 ng/ml M-CSF inducer (HY-P7050, Mce, USA). Subsequently, the cells were inoculated into sterile 6-well culture plates at 4 ml per well and incubated at 37°C for 4 days in an incubator with 5% CO_2_. The suspension cells were removed by liquid exchange and the adherent macrophages were obtained.

#### 2.3.2. Macrophage purification

Macrophages were purified using RPMI-1640 culture supplemented with 20–40% calf serum. The induced macrophages were prepared at a concentration of 2 × 10^^6^/ml. The cell suspension was then inoculated into a culture dish at a density of 2 × 10^^5^/cm^2^, and incubated at 37°C in a 5% CO_2_ incubator for 6 h. After incubation, the culture dish was gently shaken to suspend non-adherent cells, and the non-adherent cell suspension was aspirated. The adherent cells were washed 3 to 4 times with pre-warmed Hanks’ solution and then digested with an appropriate amount of calcium- and magnesium-free Hanks’ solution containing 2.5 mmol/L EDTA for 15–30 min at 37°C. The cells were separated by pipetting and centrifuged at 250 × *g* for 10 min, and the supernatant was removed. Subsequently, the cells were washed 3 to 4 times with pre-chilled Hanks’ solution, centrifuged at 250 × *g* for 10 min each time, and the supernatant was removed. Finally, the cells were resuspended in RPMI-1640 medium containing 10% calf serum.

#### 2.3.3. Macrophage identification

Purified macrophages were identified using Wright’s staining and CD14 immunofluorescence identification. Wright’s staining reagent (R-20655, Shanghai yuanye Bio-Technology, China) was used for staining, and CD14 antibody (bs-1192R, Bioss, Beijing, China) was used for immunofluorescence identification. The cells were observed under an inverted microscope (Nikon Ci-S, Japan). The macrophage polarization phenotype was determined by detecting TNF-α, IL-12, IL-10, and IL-1β in the macrophage culture supernatant using ELISA kits (ab285312, ab46035, ab185986, ab214025, Abcam).

### 2.4. Macrophage exosome extraction and identification

Macrophages were cultured in a serum-free medium (UR51101, Umibio Serum-free Media, Shanghai, China). The cell supernatant was collected when the cells reached approximately 90% confluence, and exosomes were subsequently extracted through ultracentrifugation. Firstly, the cell supernatant was centrifuged at 2000 × *g*, 4°C for 30 min (CP100MX, Hitachi, Japan) to remove cell debris. The resulting supernatant was carefully transferred to a new centrifuge tube and centrifuged again at 10,000 × *g*, 4°C for 45 min to remove larger vesicles. The supernatant from the second centrifugation step was filtered through a 0.45 μm membrane (R6BA09493, Millipore, USA), and the filtrate was collected. The filtrate was then transferred to a new centrifuge tube and subjected to ultracentrifugation 100,000 × *g*, 4°C, for 70 min using an appropriate overspeed rotor. The resulting precipitate was resuspended in 10 mL of pre-cooled 1 × PBS (E607008, Sangon Biotech, Shanghai, China) and subjected to a second round of ultracentrifuge at 100000 × *g*, 4°C, for 70 min. The precipitate obtained after the secondary ultracentrifugation step represented the extracted exosomes. It was resuspended in 350 μL of pre-cooled 1 × PBS. Subsequently, we took 20 μL of the exosome suspension for morphological observation using a transmission electron microscope (HT-7700, Hitachi, Japan). Additionally, 10 μL of the suspension was used for particle size analysis (N30E, NanoFCM, China). For protein extraction, 100 μL of the exosome suspension was used to identify the expression of the following markers: CD81 (DF2306, Affinity, USA), CD63 (DF2305, Affinity, USA), HSP70 (AF5466, Affinity, USA). The remaining exosomes were stored at −80°C.

### 2.5. Exosome micro-RNA sequencing (miRNA-seq)

Total RNA was extracted from exosomes using TRIzol reagent (Invitrogen, cat. NO 15596026) following the methods described by Chomczynski and Sacchi (1987). After RNA extraction, DNA digestion was performed using DNaseI. RNA quality was assessed by determining the A260/A280 ratio using a NanodropTM OneC spectrophotometer (Thermo Fisher Scientific Inc). Additionally, RNA integrity was confirmed by 1.5% agarose gel electrophoresis. The qualified RNAs were quantified using Qubit3.0 and the QubitTM RNA Broad Range Assay Kit (Life Technologies, Q10210). Total RNA (3 μL) was used as input for miRNA library preparation using the KC-DigitalTM small RNA Library Prep Kit for Illumina^®^ (Catalog No. DR08602, Wuhan Seqhealth Co., Ltd. China) according to the manufacturer’s instructions. The kit utilized a unique molecular identifier (UMI) of 8 random bases to label the preamplified small RNA molecules, thereby eliminating duplication bias in the PCR and sequencing steps. The eluted cDNA library was separated on a 6% PAGE gel. The ∼160 bp bands were isolated, purified, and quantified using Qubit3.0. Finally, the library was sequenced on a Hiseq X-10 sequencer (Illumina) with the PE150 model. Raw sequencing data were initially filtered using fastx_toolkit (version: 0.0.13.2) to discard low-quality reads, and adapter sequences were trimmed using cutadapt (version: 1.15). Clean reads were further processed with custom scripts to remove duplication bias introduced during library preparation and sequencing.

### 2.6. miRNA-seq data analysis

The mirdeep2 package (version: 2.0.0.8) was used to map the reads to known primary miRNA sequences in the miRBase database and predict novel miRNAs. Differential expression analysis of miRNAs between groups was conducted using the edgeR package (version: 3.12.1). A cutoff of *p*-value < 0.05 and | Log_2_Fold-change| > 1 was used to determine the statistical significance of miRNA expression differences. The target mRNAs of differentially expressed miRNAs were predicted using miRanda v3.3a and RNAhybrid (Rehmsmeier et al., 2004). Gene ontology (GO) and Kyoto Encyclopedia of genes and genomes (KEGG) enrichment analysis of the target mRNAs was performed using KOBAS software (version: 2.1.1), with a corrected *p*-value cutoff of 0.05 to identify statistically significant enrichment. The miRNA-mRNA co-expression network was constructed by WGCNA (version 1.61) with a correlation threshold >0.8 between miRNA and mRNA.

### 2.7. qRT-PCR verification

The stored miRNA samples at −80°C were reverse transcribed into cDNA using SuperScriptTM III reverse transcriptase (Invitrogen, USA), followed by real-time PCR analysis using the SYBR Green method (CloudSeq, China). The miRNA expression levels were analyzed using LC480 and calculated using the 2^^–ΔΔCt^ method, with U6 serving as the internal reference. The primer sequences for qRT-PCR are listed in Table 1.

**TABLE 1:** The primer sequences for qRT-PCR.

Common reverse primer	GTGCAGGGTCCGAGGT
cel-miR-39-RT	GTCGTATCCAGTGCAGGGTCCGAGGTATTC GCACTGGATACGACCAAGCT
cel-miR-39-F	CGCTCACCGGGTGTAAATC
U6-RT	AACGCTTCACGAATTTGCGT
U6-F	CTCGCTTCGGCAGCACA
U6-R	AACGCTTCACGAATTTGCGT
hsa-miR-125b-5p-RT	GTCGTATCCAGTGCAGGGTCCGAGGTATTC GCACTGGATACGACTCACAA
hsa-miR-125b-5p-F	CGCGTCCCTGAGACCCTAAC

F, forward primer; R, reverse primer; RT, reverse transcription.

### 2.8. Target gene network and gene enrichment analysis of miR-125b-5p

The interaction network between miR-125b-5p and its target genes was visualized using Cytoscape 3.8.2. KOBAS software (version: 2.1.1) was used to perform GO analysis and KEGG pathway enrichment analysis of the target genes, aiming to explore the potential biological functions of miR-125b-5p. A *P* < 0.05 cut-off value was used to determine statistically significant enriched pathways.

### 2.9. Statistical analysis

Statistical analysis of the data was performed using SPSS 23.0. The normality of the data was assessed using either the Shapiro-Wilk’s test or the Kolmogorov-Smirnov test. Normally distributed data were expressed as mean ± standard error, and differences between groups were evaluated using the independent samples *t*-test. For comparisons between qualitatively categorized data groups, Pearson’s χ2 or Fisher’s exact probability method was used. Receiver operating characteristic (ROC) curves were constructed to distinguish spinal tuberculosis infection from other types of spinal infections. *P* < 0.05 was considered statistically significant.

## 3. Results

### 3.1. Baseline characteristics of the patients

The baseline characteristics of the miRNA-seq samples and qRT-PCR validation samples are shown in Supplementary Table 1. In the miRNA-seq samples, all six patients were male, with three in the STB group and three in the NSTB group. All patients had received the BCG vaccine. There were no statistically significant differences in age, weight, and body mass index (BMI) between the two groups. Erythrocyte sedimentation rate (ESR) and C-reactive protein (CRP) levels were significantly higher in the STB group compared to the NSTB group. The two groups exhibited significant differences in bone mineral density (BMD), with the STB group displaying osteoporotic features. All patients in the STB group tested positive for T-SPOT.TB, while all patients in the NSTB group tested negative.

Among the qRT-PCR validation samples, there were 10 cases in the STB group and 10 cases in the NSTB group, with all patients having received the BCG vaccine. The comparison results for age, weight, BMI, ESR, CRP, and BMD between the two groups were consistent with those of the miRNA sequencing samples. Additionally, eight patients in the STB group tested positive for T-SPOT.TB, while all patients in the NSTB group tested negative.

Out of the 20 patients clinically suspected to have spinal tuberculosis preoperatively, only 14 were diagnosed with spinal tuberculosis post-operatively.

### 3.2. Macrophage characterization

**FIGURE 2:**
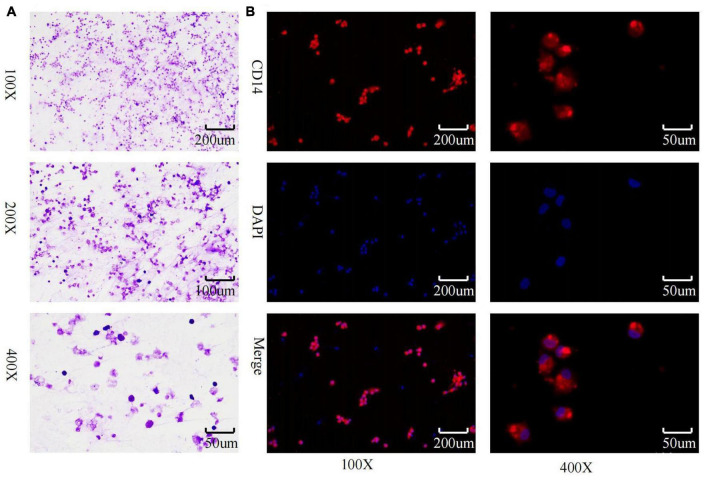
Macrophage identification. **(A)** Macrophage wright’s staining; **(B)** macrophage CD14 immunofluorescence identification and purity assessment.

**FIGURE 3:**
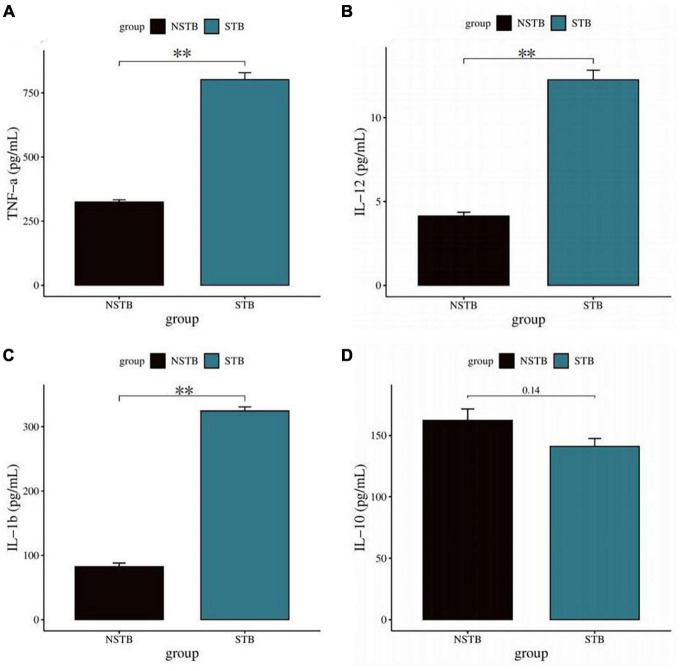
Detection of macrophage polarization phenotypes via ELISA method. **(A–C)** TNF-α, IL-12, and IL-1β levels in macrophage culture supernatants were significantly increased in the STB group; **(D)** no significant difference in IL-10 in macrophage culture supernatants between the STB and NSTB groups. **Indicates a significant difference between the two comparisons (p < 0.01).

Inverted microscopic observation of Wright’s staining of the induced macrophages revealed macrophages of various shapes, well defined with round, kidney-shaped, or irregularly shaped nuclei stained blue, and cytoplasm stained light red (Figure 2A). Immunofluorescence observation showed that the CD14-expressing part of the macrophages was stained red, while the nuclei were stained blue (Figure 2B). The positivity rate of the macrophage-specific surface antigen CD14 was over 90% for each sample. ELISA results showed high expression of TNF-α, IL-12, and IL-1β in the macrophage culture supernatants, with no significant difference in IL-10 expression levels (Figures 3A–D), indicating an M1 phenotype of macrophages in the TB-infected state.

### 3.3. Macrophage exosome characterization

**FIGURE 4:**
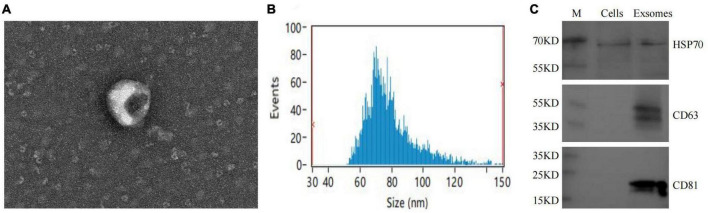
Macrophage exosomes identification. **(A)** Morphological observation of exosomes under electron microscopy; **(B)** exosome particle size analysis; **(C)** exosome-specific markers CD63, CD81, HSP70 WB identification.

The extracted macrophage exosomes were identified through electron microscopic observation, particle size analysis, and protein blotting analysis. Electron microscopic observation showed that the exosomes exhibited a disc-shaped or bowl-shaped vesicular structure without a nucleus, and their size and morphology were consistent with the characteristics of exosomes (Figure 4A). Particle size analysis showed that the exosomes were mainly between 50 and 120 nm in diameter (Figure 4B). The results of CD63, CD81, and HSP70 protein blot analysis revealed visible CD63, CD81, and HSP70 bands in the isolated macrophage exosomes (Figure 4C), further confirming that the extracted components were indeed the exosomes we intended to study.

### 3.4. Identification of exosomal differentially expressed miRNAs

**FIGURE 5:**
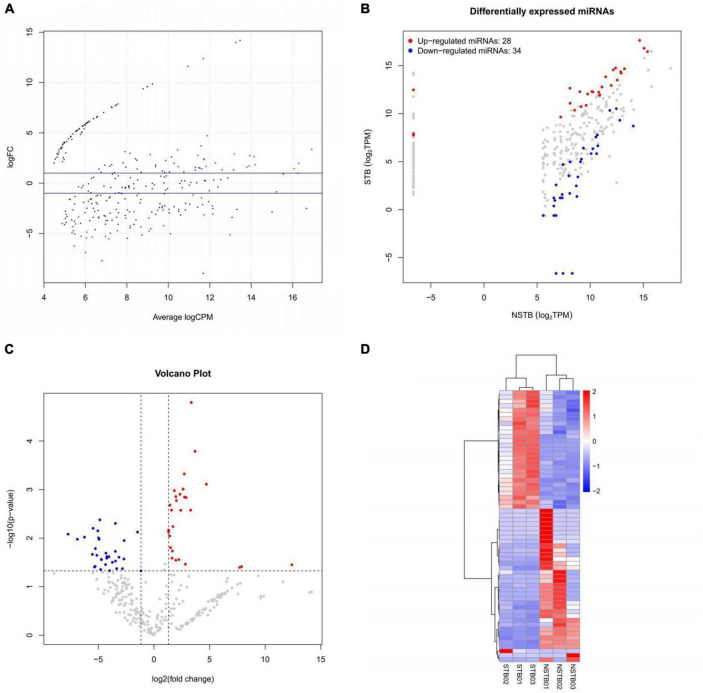
Macrophage exosome miRNAs differential expression analysis. **(A)** Differentially expressed miRNAs MA plot; **(B)** differentially expressed miRNAs volcano plot; **(C)** differentially expressed miRNAs scatter plot; **(D)** differentially expressed miRNAs hierarchical clustering heat map.

MiRNA sequencing was performed to identify differentially expressed miRNAs in macrophage exosomes between the STB (*n* = 3) and NSTB (*n* = 3) groups. A cut-off value of *P*-value < 0.05 and | Log2Fold-change| > 1 was used to determine the statistical significance of miRNA expression differences. MA plots were used to visualize the differentially expressed miRNAs, which demonstrated significant differences in miRNA expression between STB and NSTB patients (Figure 5A). Similarly, the same results were presented using volcano plots (Figure 5B). Furthermore, we represented the miRNA expression in the comparison group through a scatter plot (Figure 5C), while labeling the differentially expressed miRNAs. This analysis revealed 28 up-regulated miRNAs and 34 down-regulated miRNAs in the STB groups. The hierarchical clustering heat map of the differentially expressed miRNAs is shown in Figure 5D.

### 3.5. miRNA target gene prediction and construction of differentially expressed miRNA-mRNA interactions network

**FIGURE 6:**
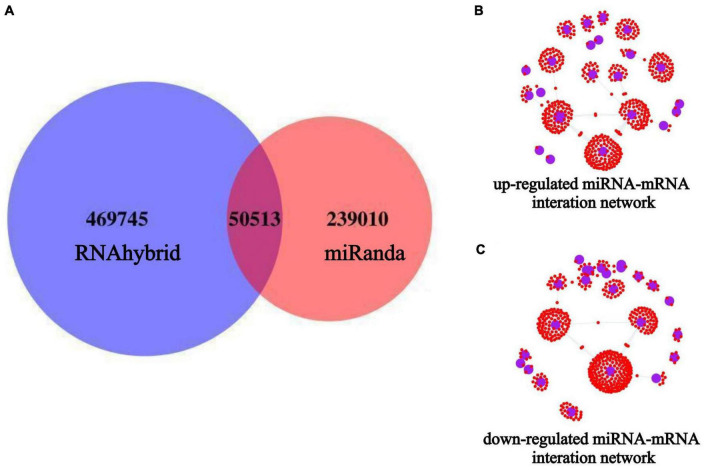
miRNAs target gene prediction and miRNA-mRNA interaction network construction. The blue circles indicate miRNAs, and the small red dots around them indicate the predicted miRNA target genes. **(A)** Target gene prediction of all miRNAs after miRNA-seq; **(B)** miRNA-mRNA interaction network of up-regulated expressed miRNAs; **(C)** miRNA-mRNA interaction network of down-regulated expressed miRNAs.

Target gene prediction was performed for all known and novel miRNAs obtained from the sequencing analysis, and the corresponding miRNA-target genes associations were identified. Among them, 289,524 target genes of miRNAs were predicted using Miranda, and 520,258 target genes of miRNAs were predicted using RNAhybrid. The final set of potential miRNA-target genes was obtained by taking the intersection of the two prediction methods, as shown in the Venn diagram (Figure 6A). Based on the results of differential miRNA analysis, we extracted the target genes of the up-and down-regulated miRNAs from the above prediction results and visualized the reciprocal relationships (Figures 6B, C). Among them, the top 5 up-regulated miRNAs in the miRNA-mRNA interaction network based on co-expression weight were novel_11, miR-24-3p, miR-125b-5p, miR-34c-5p, and miR-31-5p. The top five down-regulated miRNAs were novel_73, miR-4488, miR-328-3p, miR-339-5p, and miR-423-5p, as shown in Supplementary Table 2.

### 3.6. Target gene GO analysis and KEGG pathway enrichment analysis

**FIGURE 7:**
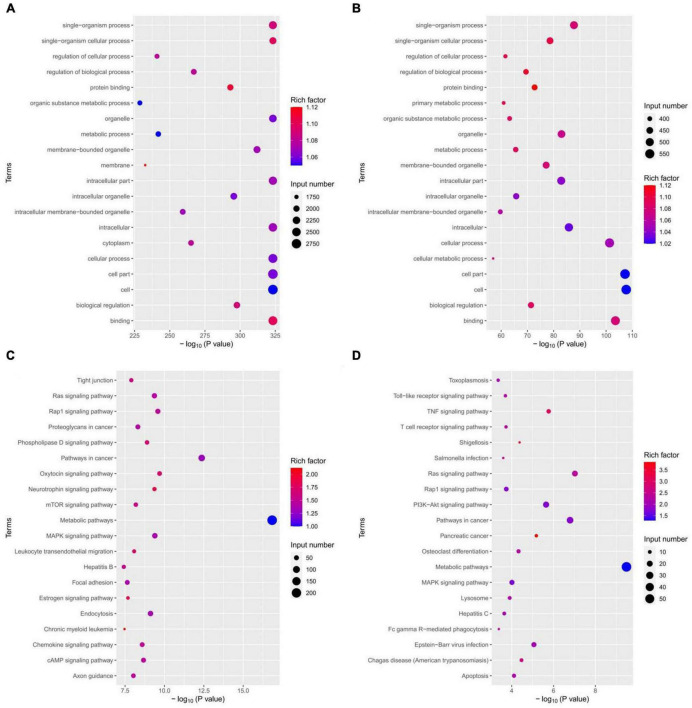
miRNAs target gene GO analysis and KEGG pathway enrichment analysis. **(A)** GO analysis of down-regulated expression of miRNA target genes; **(B)** GO analysis of up-regulated expression of miRNA target genes; **(C)** KEGG analysis of down-regulated expression of miRNA target genes; **(D)** KEGG analysis of up-regulated expression of miRNA target genes.

To gain further insight into the functions of the differentially expressed miRNA target genes, GO and KEGG analyses were performed. Figures 7A, C show the top 20 GO and KEGG terms for down-regulated expression of miRNA target genes, while Figures 7B, D present the top 20 GO and KEGG terms for up-regulated miRNA target gene expression. The GO terms encompass three types of functional annotations: biological process, cellular component, and molecular function. The GO analysis results indicated that the down-regulated and up-regulated miRNA target genes were involved in similar biological processes, cellular components, and molecular functions, although with slight differences. The KEGG pathway enrichment analysis revealed that the down-regulated miRNA target genes were associated with pathways such as the Ras signaling pathway, Rap1 signaling pathway, MAPK signaling pathway, oxytocin signaling pathway, and neurotrophin signaling pathway. On the other hand, the up-regulated miRNAs target genes were involved in pathways such as the Ras signaling pathway, Rap1 signaling pathway, MAPK signaling pathway, Toll-like receptor signaling pathway, TNF signaling pathway, T-cell receptor signaling pathway, PI3K-Akt signaling pathway, etc. This suggests that miRNAs in macrophage-derived exosomes in the tuberculosis-infected bone microenvironment may play a role in mediating the process of spinal TB. These miRNAs potentially regulate various biological processes, cellular composition, molecular functions, and signaling pathways through exosomes.

### 3.7. qRT-PCR validation and ROC curve construction

**FIGURE 8:**
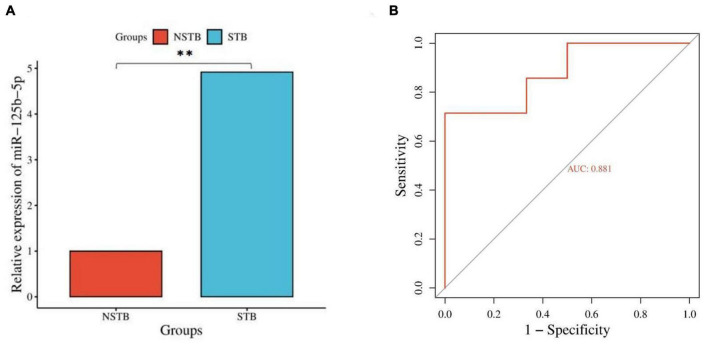
Validation and diagnostic value assessment of miR-125b-5p. **(A)** qRT-PCR validation of miR-125b-5p; **(B)** ROC curve construction for miR-125b-5p. **Indicates a significant difference between the two comparisons (p < 0.01).

Based on the results of differentially expressed miRNAs, we selected miR-125b-5p for further qRT-PCR validation, considering its fold change, *p*-value, and false discovery rate (FDR). The miR-125b-5p expression was up-regulated in bone marrow blood samples from patients with spinal TB compared to those without spinal TB (Figure 8A). The qRT-PCR results were consistent with the findings from high-throughput sequencing. To assess the diagnostic value of miR-125b-5p for spinal TB, miR-125b-5p levels were measured in bone marrow blood samples from 20 patients with clinically suspected spinal TB, and a ROC curve was constructed (Figure 8B). The area under the ROC curve (AUC) was 0.881, with a 95% confidence interval of 0.729∼1.000. The results suggest that miR-125b-5p in macrophage-derived exosomes from the tuberculosis-infected bone microenvironment could serve as a potential and valuable biomarker for spinal tuberculosis.

### 3.8. miR-125b-5p target gene network and gene enrichment analysis

**FIGURE 9:**
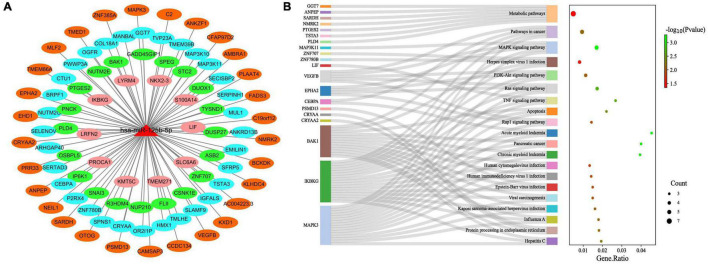
Functional studies of miR-125b-5p target genes. **(A)** miR-125b-5p target gene network; **(B)** miR-125b-5p target gene KEGG pathway enrichment analysis.

We further constructed miR-125b-5p target gene subnetworks to demonstrate the interaction between miR-125b-5p and its target genes (Figure 9A). Additionally, gene enrichment analysis was performed to explore the potential functions of miR-125b-5p (Figure 9B). KEGG pathway enrichment analysis revealed that miR-125b-5p was involved in a total of 56 signaling pathways, human diseases, or pathological conditions. Notably, the MAPK, TNF, Ras, Rap1, and PI3K-Akt signaling pathways were found to be important in the regulation of the spinal tuberculosis disease course mediated by miR-125b-5p.

## 4. Discussion

Tuberculosis remains one of the most significant global public health challenges. Spinal TB, characterized by its insidious nature and challenging early stage diagnosis, poses a high risk as the disease progresses. Traditional anti-tuberculosis drug therapy often proves ineffective in controlling spinal TB due to the emergence of drug-resistant tuberculosis bacilli. This leads to disease relapse and imposes severe consequences on patients while consuming substantial medical resources (World Health Organization [WHO], 2020).

During MTB infection, macrophages interact with pathogens to create a specific immune milieu (Bo et al., 2023). Macrophages are the major host cells for MTB and can polarize into the M1 phenotype, resulting in the production of large amounts of pro-inflammatory cytokines such as tumor necrosis factor-α (TNF-α), interleukin-1 (IL-1), and interleukin-6 (IL-6) (Cooper et al., 2011). This pro-inflammatory environment, in which exosomes play a key role, helps combat the pathogen (Bhatnagar et al., 2007). Studies have shown that exosomes derived from MTB-infected macrophages can regulate immune function by transferring signaling molecules to recipient cells (Giri and Schorey, 2008; Singh et al., 2012). However, it is important to note that macrophage exosomes exhibit both beneficial and detrimental roles. Evidence suggests that macrophage exosomes are involved in the immune evasion pathway of MTB infection, enabling MTB to infect more host cells (Sun Y. et al., 2021). Therefore, it is of paramount importance to investigate the role of macrophage exosomes in disease progression following TB infection.

*Mycobacterium* tuberculosis -infected macrophages release a significant number of exosomes containing pathogen-associated molecular patterns (PAMPs) that activate the inflammatory response and regulate immune function in the body (Bhatnagar et al., 2007). Interestingly, exosomes from infected macrophages can activate uninfected macrophages and recruit other immune cells. A study demonstrated that intervention with exosomes isolated from mycobacteria-infected mice or cultured significantly up-regulated cytokine expression levels in mouse bone marrow macrophages. Moreover, these exosomes enhanced the migration of CFSE-labeled macrophages and spleen cells, and intranasal administration of these exosomes promoted the recruitment of CD 11b^+^ cells to the lung (Singh et al., 2012). Additional studies have revealed that exosomes released from *Mycobacterium* tuberculosis-infected cells can inhibit the IFN-γ-mediated activation of naive macrophages, thereby mediating immune escape (Singh et al., 2011). Furthermore, exosomal RNA from MTB-infected cells can stimulate higher levels of cytokines and chemokines, leading to increased macrophage apoptosis (Singh et al., 2015).

MiRNAs are important mediator molecules for exosomes to perform their functions. High-throughput miRNA sequencing has greatly accelerated the exploration of expression levels and biological functions of exosomal miRNAs in various diseases. Notably, miRNAs have been found to play an important regulatory role in Mtb-infected macrophages, exhibiting significant differential expression of certain miRNAs following Mtb infection. These changes in miRNA expression are involved in multiple immune responses of macrophages against MTB (Mirzaei et al., 2021). A study by Zhang et al. (2019) demonstrated that down-regulating miR-20b-5p promoted the survival of *Mycobacterium* tuberculosis in RAW 264.7 macrophages by attenuating apoptosis through up-regulating Mcl-1. Furthermore, the diagnostic potential and application of miRNAs as biomarkers in TB have been demonstrated in numerous studies. Serum miRNA levels in TB patients have shown promise in aiding the detection of early stage TB (Miotto et al., 2013). Other studies have highlighted the potential of miRNA 144 and miRNA-185-5p in TB diagnosis (Liu et al., 2011; Kaushik et al., 2021), whereas miRNA-889 may serve as a potential diagnostic marker for latent TB infection (Chen et al., 2020). However, current research on the role of miRNAs in tuberculosis has predominantly focused on pulmonary tuberculosis infection, with limited studies on extrapulmonary tuberculosis. Yang et al. (2019) revealed that miR-155 was down-regulated in spinal tuberculosis and could potentially mediate disc destruction by regulating the expression levels of related proteins in the intervertebral disc through up-regulating MMP13 expression. Liang et al. (2023) successfully developed a diagnostic model based on a 3-plasma miRNA biomarker signature (hsa-miR-506-3p, hsa-miR 543, hsa-miR-195-5p) and suggested its potential use in differentiating spinal tuberculosis from other spinal destructive diseases and tuberculosis.

In this study, we investigated for the first time the miRNA expression levels in macrophage-derived exosomes from the tuberculosis-infected bone microenvironment using high-throughput sequencing. We successfully constructed miRNA differential expression profiles and identified miR-125b-5p as a biomarker for spinal tuberculosis, which was further validated by qRT-PCR. Previous research has shown that MiR-125b-5p is significantly up-regulated in monocyte-macrophages of TB patients, directly associated with macrophage apoptosis and the inflammatory response induced by TB infection (Liu G. et al., 2020). Similar results have been reported in Vγ2Vδ2 T cells from TB patients (Shen et al., 2017). Rajaram et al. (2011) demonstrated that miR-125b was up-regulated in MTB-stimulated macrophages, targeting the 3′ UTR of the TNF transcript and down-regulating TNF production. Our findings are consistent with these studies, indicating high expression of miR-125b-5p in various immune cells, primarily monocytes-macrophages, after TB infection. However, it is noteworthy that our results show an opposite trend to those of Zhou et al. (2016) and Sun X. et al. (2021), which may be attributed to the difference between bone marrow blood exosomes and circulating exosomes, as well as variations in the disease mechanisms of different types of diseases. However, the exact reason for this contrasting trend remains uncertain.

The ROC curve further confirmed the diagnostic value of miR-125b-5p in spinal TB. Additionally, bioinformatics-based gene function mining revealed the therapeutic potential of miR-125b-5p as a therapeutic target for intervention in spinal TB. The results of the KEGG pathway enrichment analysis, which focused on the target genes of miR-125b-5p, highlighted the importance of investigating the functional relationships between the MAPK, TNF, Ras, Rap1, PI3K-Akt signaling pathways and miR-125b-5p in spinal TB.

## 5. Conclusion

This study demonstrates differential expression of miRNAs in macrophage-derived exosomes from the tuberculosis-infected bone microenvironment. Notably, miRNA-125b-5p shows significant up-regulation in spinal tuberculosis and holds potential as a diagnostic biomarker for spinal tuberculosis.

## Data availability statement

The data presented in the study are deposited in the SRA repository with a BioProject accession number: PRJNA997564.

## Ethics statement

The studies involving human participants were reviewed and approved by the Ethics Committee of Xiangya Hospital. The patients/participants provided their written informed consent to participate in this study.

## Author contributions

ZS: conceptualization, software, data curation, visualization, and writing–original draft. XP, XW, and HZ: conceptualization, project administration, validation, writing–review and editing, and funding acquisition. All authors contributed to the article and approved the submitted version.

## References

[B1] Alipoor S. Mortaz E. Tabarsi P. Farnia P. Mirsaeidi M. Garssen J. (2017). Bovis Bacillus Calmette-Guerin (BCG) infection induces exosomal miRNA release by human macrophages. *J. Transl. Med.* 15:105. 10.1186/s12967-017-1205-9 28499455 PMC5427544

[B2] Alipoor S. Tabarsi P. Varahram M. Movassaghi M. Dizaji M. Folkerts G. (2019). Serum exosomal miRNAs are associated with active pulmonary tuberculosis. *Dis. Markers* 2019:1907426. 10.1155/2019/1907426 30886653 PMC6388314

[B3] Bhatnagar S. Shinagawa K. Castellino F. Schorey J. (2007). Exosomes released from macrophages infected with intracellular pathogens stimulate a proinflammatory response in vitro and in vivo. *Blood* 110 3234–3244. 10.1182/blood-2007-03-079152 17666571 PMC2200902

[B4] Bo H. Moure U. Yang Y. Pan J. Li L. Wang M. (2023). *Mycobacterium tuberculosis*-macrophage interaction: Molecular updates. *Front. Cell Infect. Microbiol.* 13:1062963. 10.3389/fcimb.2023.1062963 36936766 PMC10020944

[B5] Chen D. Chen Y. Lin C. Lo C. Liu H. Liao T. (2020). MicroRNA-889 inhibits autophagy to maintain mycobacterial survival in patients with latent tuberculosis infection by targeting TWEAK. *mBio* 11:e03045-19. 10.1128/mBio.03045-19 31992621 PMC6989109

[B6] Chomczynski P. Sacchi N. (1987). Single-step method of RNA isolation by acid guanidinium thiocyanate-phenol-chloroform extraction. *Anal. Biochem.* 162 156–159. 10.1006/abio.1987.9999 2440339

[B7] Cooper A. Mayer-Barber K. Sher A. (2011). Role of innate cytokines in mycobacterial infection. *Mucosal Immunol.* 4 252–260. 10.1038/mi.2011.13 21430655 PMC3294290

[B8] Cui J. Chen G. Wen D. Wang Y. Zhao Z. Wu C. (2020). Asap1 affects the susceptibility of Zebrafish to *Mycobacterium* by regulating macrophage migration. *Front. Cell Infect. Microbiol.* 10:519503. 10.3389/fcimb.2020.519503 33194781 PMC7658321

[B9] Genoula M. Marín Franco J. Maio M. Dolotowicz B. Ferreyra M. Milillo M. (2020). Fatty acid oxidation of alternatively activated macrophages prevents foam cell formation, but *Mycobacterium tuberculosis* counteracts this process via HIF-1α activation. *PLoS Pathog.* 16:e1008929. 10.1371/journal.ppat.1008929 33002063 PMC7553279

[B10] Giri P. Schorey J. (2008). Exosomes derived from M. Bovis BCG infected macrophages activate antigen-specific CD4+ and CD8+ T cells in vitro and in vivo. *PLoS One* 3:e2461. 10.1371/journal.pone.0002461 18560543 PMC2413420

[B11] Imamiya R. Shinohara A. Yakura D. Yamaguchi T. Ueda K. Oguro A. (2023). *Escherichia coli*-derived outer membrane vesicles relay inflammatory responses to macrophage-derived exosomes. *mBio* 14:e03051-22. 10.1128/mbio.03051-22 36648227 PMC9973271

[B12] Kaushik A. Wu Q. Lin L. Li H. Zhao L. Wen Z. (2021). Exosomal ncRNAs profiling of mycobacterial infection identified miRNA-185-5p as a novel biomarker for tuberculosis. *Brief. Bioinform.* 22:bbab210. 10.1093/bib/bbab210 34169968

[B13] Khanna K. Sabharwal S. (2019). Spinal tuberculosis: A comprehensive review for the modern spine surgeon. *Spine J.* 19 1858–1870. 10.1016/j.spinee.2019.05.002 31102727

[B14] Liang Q. Jin W. Huang Z. Yin H. Liu S. Liu L. (2023). A plasma 3-marker microRNA biosignature distinguishes spinal tuberculosis from other spinal destructive diseases and pulmonary tuberculosis. *Fron.t Cell Infect. Microbiol.* 13:1125946. 10.3389/fcimb.2023.1125946 36926516 PMC10011472

[B15] Liu G. Wan Q. Li J. Hu X. Gu X. Xu S. (2020). Silencing miR-125b-5p attenuates inflammatory response and apoptosis inhibition in *Mycobacterium tuberculosis*-infected human macrophages by targeting DNA damage-regulated autophagy modulator 2 (DRAM2). *Cell Cycle* 19 3182–3194. 10.1080/15384101.2020.1838792 33121314 PMC7714508

[B16] Liu Y. Wang X. Jiang J. Cao Z. Yang B. Cheng X. (2011). Modulation of T cell cytokine production by miR-144* with elevated expression in patients with pulmonary tuberculosis. *Mol. Immunol.* 48 1084–1090. 10.1016/j.molimm.2011.02.001 21367459

[B17] Liu Y. Wang Y. Lv Q. Li X. (2020). Exosomes: From garbage bins to translational medicine. *Int. J. Pharm.* 583:119333. 10.1016/j.ijpharm.2020.119333 32348800

[B18] Lyu L. Zhang X. Li C. Yang T. Wang J. Pan L. (2019). Small RNA profiles of serum exosomes derived from individuals with latent and active tuberculosis. *Front. Microbiol.* 10:1174. 10.3389/fmicb.2019.01174 31191492 PMC6546874

[B19] Miotto P. Mwangoka G. Valente I. C. Norbis L. Sotgiu G. Bosu R. (2013). miRNA signatures in sera of patients with active pulmonary tuberculosis. *PLoS One* 8:e80149. 10.1371/journal.pone.0080149 24278252 PMC3836984

[B20] Mirzaei R. Babakhani S. Ajorloo P. Ahmadi R. Hosseini-Fard S. Keyvani H. (2021). The emerging role of exosomal miRNAs as a diagnostic and therapeutic biomarker in *Mycobacterium tuberculosis* infection. *Mol. Med.* 27:34. 10.1186/s10020-021-00296-1 33794771 PMC8017856

[B21] Pang Y. An J. Shu W. Huo F. Chu N. Gao M. (2019). Epidemiology of extrapulmonary tuberculosis among inpatients. China, 2008-2017. *Emerg. Infect. Dis.* 25 457–464. 10.3201/eid2503.180572 30789144 PMC6390737

[B22] Rajaram M. Ni B. Morris J. Brooks M. Carlson T. Bakthavachalu B. (2011). *Mycobacterium tuberculosis* lipomannan blocks TNF biosynthesis by regulating macrophage MAPK-activated protein kinase 2 (MK2) and microRNA miR-125b. *Proc. Natl. Acad. Sci. U.S.A.* 108 17408–17413. 10.1073/pnas.1112660108 21969554 PMC3198317

[B23] Rehmsmeier M. Steffen P. Hochsmann M. Giegerich R. (2004). Fast and effective prediction of microRNA/target duplexes. *RNA* 10 1507–1517. 10.1261/rna.5248604 15383676 PMC1370637

[B24] Sachdeva K. Goel M. Sudhakar M. Mehta M. Raju R. Raman K. (2020). *Mycobacterium tuberculosis* (Mtb) lipid mediated lysosomal rewiring in infected macrophages modulates intracellular Mtb trafficking and survival. *J. Biol. Chem.* 295 9192–9210. 10.1074/jbc.RA120.012809 32424041 PMC7335774

[B25] Shen H. Gu J. Xiao H. Liang S. Yang E. Yang R. (2017). Selective destruction of interleukin 23-induced expansion of a major antigen-specific γδ T-cell subset in patients with tuberculosis. *J. Infect. Dis.* 215 420–430. 10.1093/infdis/jiw511 27789724 PMC5853380

[B26] Singh P. LeMaire C. Tan J. Zeng E. Schorey J. (2011). Exosomes released from *M. tuberculosis* infected cells can suppress IFN-γ mediated activation of naïve macrophages. *PLoS One* 6:e18564. 10.1371/journal.pone.0018564 21533172 PMC3077381

[B27] Singh P. Li L. Schorey J. (2015). Exosomal RNA from *Mycobacterium tuberculosis*-infected cells is functional in recipient macrophages. *Traffic* 16 555–571. 10.1111/tra.12278 25753779 PMC5735426

[B28] Singh P. Smith V. Karakousis P. Schorey J. (2012). Exosomes isolated from mycobacteria-infected mice or cultured macrophages can recruit and activate immune cells in vitro and in vivo. *J. Immunol.* 189 777–785. 10.4049/jimmunol.1103638 22723519 PMC3685416

[B29] Smith V. Cheng Y. Bryant B. Schorey J. (2017). Exosomes function in antigen presentation during an in vivo *Mycobacterium tuberculosis* infection. *Sci. Rep.* 7:43578. 10.1038/srep43578 28262829 PMC5338015

[B30] Sun X. Liu K. Wang X. Zhang T. Li X. Zhao Y. (2021). Diagnostic value of microRNA-125b in peripheral blood mononuclear cells for pulmonary tuberculosis. *Mol. Med. Rep.* 23:249. 10.3892/mmr.2021.11888 33537800

[B31] Sun Y. Pi J. Xu J. (2021). Emerging role of exosomes in tuberculosis: From immunity regulations to vaccine and immunotherapy. *Front. Immunol.* 12:628973. 10.3389/fimmu.2021.628973 33868247 PMC8047325

[B32] World Health Organization [WHO] (2020). *Global tuberculosis report 2020.* Geneva: World Health Organization.

[B33] Yang C. Shi Z. Hu J. Wei R. Yue G. Zhou D. (2019). miRNA-155 expression and role in pathogenesis in spinal tuberculosis-induced intervertebral disc destruction. *Exp. Ther. Med.* 17 3239–3246. 10.3892/etm.2019.7313 30936999 PMC6434382

[B34] Zhang D. Yi Z. Fu Y. (2019). Downregulation of miR-20b-5p facilitates *Mycobacterium tuberculosis* survival in RAW 264.7 macrophages via attenuating the cell apoptosis by Mcl-1 up-regulation. *J. Cell Biochem.* 120 5889–5896. 10.1002/jcb.27874 30378171

[B35] Zhou M. Yu G. Yang X. Zhu C. Zhang Z. Zhan X. (2016). Circulating microRNAs as biomarkers for the early diagnosis of childhood tuberculosis infection. *Mol. Med. Rep.* 13 4620–4626. 10.3892/mmr.2016.5097 27082104 PMC4878571

