# Supplementary material for: Differential expression analysis of miRNAs in macrophage-derived exosomes in the tuberculosis-infected bone microenvironment
Source: Front Microbiol. 2023 Aug 3;14:1236012. doi: 10.3389/fmicb.2023.1236012 (PMC10435735; doi:10.3389/fmicb.2023.1236012)
Supplement: Supplementary file 1 [file Table_1.docx]

Table 1: Baseline characteristics of miRNA sequencing samples and qRT-PCR validation samples.

|  |  | miRNA-seq samples | | | qRT-PCR validation samples | | |
| --- | --- | --- | --- | --- | --- | --- | --- |
|  |  | STB(n=3) | NSTB(n=3) | P value | STB(n=10) | NSTB(n=10) | P value |
| Gender | Male | 3 | 3 | / | 5 | 5 | / |
|  | Female | 0 | 0 |  | 5 | 5 |  |
| Age (y) | | 33.33±4.16 | 35.33±1.53 | 0.478 | 31.90±4.68 | 33.10±3.00 | 0.503 |
| Weight (kg) | | 64.33±4.04 | 66.67±1.53 | 0.403 | 55.60±7.63 | 56.70±8.90 | 0.770 |
| BMI (kg/m^2^) | | 21.56±0.73 | 22.46±0.81 | 0.228 | 19.78±1.43 | 20.36±1.92 | 0.451 |
| ESR (mm/h) | | 35.33±8.62 | 6.66±1.15 | 0.005 | 45.00±15.37 | 7.30±2.21 | 0.000 |
| CRP (mg/L) | | 24.17±7.89 | 3.17±0.55 | 0.010 | 26.28±9.78 | 3.98±1.08 | 0.000 |
| BMD T-score | | -2.77±0.58 | 0.23±0.67 | 0.001 | -2.68±0.30 | 0.05±0.81 | 0.000 |
| T-SPOT.TB positve rate | | 3/3 | 0/3 | / | 8/10 | 0/10 | 0.001 |
| BCG vaccination | | 3/3 | 3/3 | / | 10/10 | 10/10 | / |
