# Supplementary material for: Differential expression analysis of miRNAs in macrophage-derived exosomes in the tuberculosis-infected bone microenvironment
Source: Front Microbiol. 2023 Aug 3;14:1236012. doi: 10.3389/fmicb.2023.1236012 (PMC10435735; doi:10.3389/fmicb.2023.1236012)
Supplement: Supplementary file 2 [file Table_2.docx]

Table 2: Characteristics of up-/down-regulated miRNAs with top 5 co-expression weight value in miRNA-mRNA interaction network.

| miRNA ID | up-/down- regulation | miRNA Sequence | Target gene number | Log_2_ FC | P value | FDR |
| --- | --- | --- | --- | --- | --- | --- |
| novel_11 | up-regulation | AGCUCUGGGGAUUGGCAA | 283 | 12.40 | 0.035 | 0.200 |
| miR-24-3p | up-regulation | UGGCUCAGUUCAGCAGGAACAG | 139 | 1.49 | 0.016 | 0.145 |
| miR-125b-5p | up-regulation | UCCCUGAGACCCUAACUUGUGA | 91 | 3.36 | <0.001 | 0.005 |
| miR-34c-5p | up-regulation | AGGCAGUGUAGUUAGCUGAUUGC | 38 | 4.72 | 0.001 | 0.045 |
| miR-31-5p | up-regulation | AGGCAAGAUGCUGGCAUAGCU | 35 | 3.70 | <0.001 | 0.025 |
| novel_73 | down-regulation | GGCGGGGCCCGGGGGGCG | 1013 | -5.53 | 0.022 | 0.179 |
| miR-4488 | down-regulation | AGGGGGCGGGCUCCGGCG | 713 | -4.04 | 0.024 | 0.181 |
| miR-328-3p | down-regulation | CUGGCCCUCUCUGCCCUUCCGU | 665 | -3.45 | 0.030 | 0.185 |
| miR-339-5p | down-regulation | UCCCUGUCCUCCAGGAGCUCACG | 459 | -2.83 | 0.042 | 0.217 |
| miR-423-5p | down-regulation | UGAGGGGCAGAGAGCGAGACUUU | 370 | -3.47 | 0.019 | 0.164 |

Log_2_ FC: Log value of Fold Change.

FDR: False Discovery Rate.
